# A triplex crystal digital PCR for the detection of genotypes I and II African swine fever virus

**DOI:** 10.3389/fvets.2024.1351596

**Published:** 2024-04-02

**Authors:** Kaichuang Shi, Xinxiu Qian, Yuwen Shi, Haina Wei, Yi Pan, Feng Long, Qingan Zhou, Shenglan Mo, Liping Hu, Zongqiang Li

**Affiliations:** ^1^School of Basic Medical Sciences, Youjiang Medical University for Nationalities, Baise, China; ^2^College of Animal Science and Technology, Guangxi University, Nanning, China; ^3^Guangxi Center for Animal Disease Control and Prevention, Nanning, China

**Keywords:** African swine fever virus (ASFV), multiplex crystal digital PCR (cdPCR), co-infection, genotype I, genotype II

## Abstract

African swine fever (ASF) is a highly contagious and lethal viral disease that causes severe hemorrhagic fever in pigs. It keeps spreading around the world, posing a severe socioeconomic risk and endangering biodiversity and domestic food security. ASF first outbroke in China in 2018, and has spread to most provinces nationwide. Genotypes I and II ASF virus (ASFV) as the etiological pathogens have been found in China. In this study, three pairs of specific primers and probes targeting the ASFV B646L gene, F1055L gene, and E183L gene were designed to detect universal, genotype I, and genotype II strains, respectively. A triplex crystal digital PCR (cdPCR) was established on the basis of optimizing various reaction conditions. The assay demonstrated remarkably sensitive with low limits of detection (LODs) of 5.120, 4.218, 4.588 copies/reaction for B646L, F1055L, and E183L gene, respectively; excellent repeatability with 1.24–2.01% intra-assay coefficients of variation (CVs) and 1.32–2.53% inter-assay CVs; good specificity for only detection of genotypes I and II ASFV, without cross-reactivity with PCV2, PRV, SIV, PRRSV, PEDV, FMDV, and CSFV. The triplex cdPCR was used to test 1,275 clinical samples from Guangxi province of China, and the positivity rates were 5.05, 3.22, and 1.02% for genotype I, genotype II, and co-infection of genotypes I and II, respectively. These 1,275 clinical samples were also detected using a reported reference triplex real-time quantitative PCR (qPCR), and the agreements of detection results between these two methods were more than 98.98%. In conclusion, the developed triplex cdPCR could be used as a rapid, sensitive, and accurate method to detect and differentiate genotypes I and II strains of ASFV.

## 1 Introduction

African swine fever (ASF) is a highly contagious and lethal infectious disease of pigs. ASF virus (ASFV), the etiological pathogen, belongs to the *Asfivirus* genus in the *Asfiviridae* family. ASFV is a double-stranded, linear DNA virus, with a genome of 170–193 kb in length, and contains 151–167 open reading frames (ORFs) encoding more than 168 structural and nonstructural proteins (1). ASF is an acute, highly infectious, and deadly illness to domestic pigs and wild boars, with a fatality rate of as high as 100% (2). The typical symptoms and pathological changes of ASF are characterized by deadly hemorrhagic fever, respiratory distress, skin cyanosis, thrombocytopenia, and extensive bleeding from the kidneys, gastrointestinal mucosa, lymph nodes, and other organs (3, 4). ASF was first discovered in Kenya of Africa in 1921, and since then, ASF has disseminated across various countries of sub-Saharan Africa, and spread to other continents outside Africa (5, 6). The first outbreak of ASF in China was discovered in 2018 in Jilin province, Northeastern China, then quickly spread to almost all provinces in the country within a year, which causing a devastating blow to China’s pig industry (7–9). Since 2018, this highly contagious disease has spread rapidly to many countries in Southeastern Asia, and America (5, 10–12). To date, ASF has been discovered in many countries, mainly in Africa, Asia, Europe, and the American Caribbean (13, 14), and has seriously damaged the pig industry worldwide. The history of prevention and control to ASF in various countries shows that, as the absence of a specific vaccine and effective measures, the epidemic of ASF, once introduced, is very difficult to eradicate in a short time (15). As for China, ASF is currently the top priority disease for prevention and control.

ASFV can be categorized into 24 genotypes on the basis of ASFV B646L gene’s 3′ end sequence (16, 17). Outside of Africa, only genotypes I and II strains of ASFV have been discovered (6, 17, 18). In China, genotype II ASFV was first identified in 2018, and has been the predominant strains circulating in the field thereafter (19, 20). Genotype I ASFV was first discovered in 2020, and has been confirmed in Henan, Shandong, and Guangxi provinces in China (19, 21–23), but its prevalence and harm throughout the country require further investigation and evaluation. Recently, the naturally recombinant strain of genotype I and II was discovered in China, and showed highly virulent and lethal to domestic pigs (24). Nowadays, genotype I, genotype II, and genotype I and II recombinant strains of ASFV are prevalent simultaneously in China, and co-infections of genotype I and II have been reported (19, 23). These situations increase the complexity of epidemic strains and increase the difficulty of prevention and control, which inflicts significant harm on the China’s pig industry (8, 9, 25). Therefore, rapid, and accurate detection and identification of the circulating ASFV genotype is of utmost importance for implementing effective prevention and control measures in the early infected stage.

The real-time quantitative PCR (qPCR) has been widely used to detect viral nucleic acids in many laboratories. This advanced molecular technique has garnered significant recognition and acceptance due to its advantages of low chance of contamination, excellent accuracy, superior sensitivity, exceptional specificity, convenience, and efficiency (26, 27). However, the disadvantages of the qPCR mainly include the fluctuation of Ct values depending on threshold setting, the high sensitivity to reaction inhibitors, and the complex procedure of generating calibration curves, which limits the application of qPCR. Therefore, the digital PCR (dPCR) is a new and better choice for detection of low copies of viral nucleic acids. The dPCR is an emerging technology in the field of microbiology, and has the main advantages of high specificity and sensitivity, excellent repeatability, the ability to achieve absolute quantification without the requirement of a reference gene, Ct value and standard curve, and strong tolerance to PCR inhibitors (28, 29). The dPCR can be divided into crystal digital PCR (cdPCR) and droplet digital PCR (ddPCR) (30, 31). Several reports have established the dPCR to detect ASFV (32–36), but no multiplex dPCR to simultaneously detect genotype I and genotype II ASFV has ever been reported. Here, a triplex cdPCR was developed to detect and differentiate genotype I and genotype II ASFV, and used to test 1,275 clinical samples to validate its applicability in the field.

## 2 Materials and methods

### 2.1 Collection of clinical samples

From March 2023 to August 2023, a total of 1,275 clinical samples (including lung, spleen, kidney, tonsil, and lymph nodes from each pig) were obtained from 1,275 dead pigs from different 4 pig farms, 6 harmless treatment plants, and 17 slaughterhouses in Guangxi province of Southern China. The dead pigs showed different manifestations such as fever, diarrhea, cough, and/or redness, cyanosis, or bleeding on skin. The written consent of the animal owners was obtained, and the research group promised not to disclose the detailed information about the relevant pig farms and the incidence of diseases in the pig herds in this study. These tissues were transported under ≤4°C within 12 h to the laboratory, and stored at −80°C until used.

### 2.2 Obtainment of virus strains

The foot-and-mouth disease virus (FMDV, strain O/Mya98/XJ/2010), swine influenza virus (SIV, strain TJ), porcine reproductive and respiratory syndrome virus (PRRSV, strain JXA1-R), classical swine fever virus (CSFV, strain C), porcine epidemic diarrheal virus (PEDV, strain CV777), pseudorabies virus (PRV, strain Bartha-K61), and porcine circovirus type 2 (PCV2, strain ZJ/C) were obtained from commercial company (Huapai Co., Ltd., Chengdu, China). The genotypes I and II ASFV clinical samples were obtained from Guangxi Center for Animal Disease Control and Prevention (CADC), China.

### 2.3 Design of primers and probes

Three sets of specific primers and probes were designed as described by Qian et al. in the previous report (23). The primers and probe targeting the B646L gene were used to detect the 24 different ASFV genotypes, those targeting the F1055L gene were used to specifically detect ASFV genotype I, and those targeting the E183L gene were used to specifically detect ASFV genotype II. The primers and probes are shown in Table 1.

**Table 1:** The used primers and probes.

Name	Sequence (5′ → 3′)	Tm/°C	Product/bp
B646L-F	CAAAGTTCTGCAGCTCTTACA	56.0	120
B646L-R	TGGGTTGGTATTCCTCCCGT	61.6	
B646L-P	FAM-TCCGGGYGCGATGATGATTACCTT-BHQ1	63.1	
F1055L-F	GCAGGTAGTTTGATTCCCTT	56.0	122
F1055L-R	GGGCGATGTCTCTGTAAGT	57.6	
F1055L-P	VIC-TGAGACAGCAGATTAAGCAGAGCCCCTG-BHQ1	67.4	
E183L-F	CGCGAGTGCTCCTGCTC	60.1	133
E183L-R	GGAGTTTTCTAGGTCTTTATGCGT	57.6	
E183L-P	CY5-TTACACGACAGTCACTACTCAGAACACTGC-BHQ2	64.0	

### 2.4 Extraction of nucleic acids

The clinical tissues were homogenized, freeze-thawed, vortexed, and centrifuged as described by Qian et al. (23). Total nucleic acids were extracted from 200 μL supernatants using the GeneRotex 96 Automated Nucleic Acid Extractor and the Viral DNA/RNA Isolation Kit 4.0 (TIANLONG, Xian, China) according to the manufacturer’s constructions, and stored at −80°C until used.

### 2.5 Generation of the standard plasmid constructs

The standard plasmid constructs were generated according to Qian et al. (23) with minor modification. Total nucleic acids from ASFV-positive samples were used as templates to amplify the targeted fragments by PCR using the designed primers (Table 1). The amplification products were purified using MiniBEST DNA Fragment Purification Kit Ver.4.0 (TaKaRa, Dalian, China), cloned into the pMD18-T vector (TaKaRa, Dalian, China), then transformed into *E. coli* DH5α cells (TaKaRa, Dalian, China). The positive clones were cultured at 37°C for 22–24 h, and the recombinant standard plasmid constructs were extracted using MiniBEST Plasmid Extraction Kit Ver.5.0 (TaKaRa, Dalian, China). The plasmids were sent to IGE biotechnology LTD (Guangzhou, China) for sequencing using Sanger sequencing method, and the sequences of inserted fragments were confirmed by BLAST analysis at the National Center for Biotechnology Information (NCBI).^1^ Then, the correct plasmid constructs were named p-dASFV-B646L, p-dASFV-F1055L, and p-dASFV-E183L, respectively, and used to established the triplex cdPCR.

The standard plasmid constructs were measured using the NanoDrop spectrophotometer (Thermo Fisher, Waltham, MA, USA) 260 nm and 280 nm, and determined their concentrations using the formula as follows: copy number (copies/μL) = (plasmid construct concentration × 10^−9^ × 6.02 × 10^23^)/ (660 Dalton/bases × DNA length).

### 2.6 Determination of reaction conditions

The Naica™ Sapphire Crystal System (Stilla Technologies™, Villejuif, France) was used to optimize various parameters of the cdPCR, i.e., the annealing temperature, reaction cycles, and the primer and probe concentrations. The triplex cdPCR reaction system was set at 25 μL. These conditions were optimized to maximize the accuracy and reliability of the reaction for precise and sensitive detection of the targeted analytes. The processes of the triplex cdPCR, including preparation of the Sapphire Chips, partition and PCR amplification, and acquisition of three-color fluorescence images and analysis of the droplet crystals, were performed according to the operation manual provided by the manufacturer (Stilla Technologies™, Villejuif, France).

### 2.7 Generation of the standard curves

To generate the standard curves, three standard plasmid constructs were mixed together and 10-fold serially diluted, and the mixtures with final reaction concentrations ranging from 1.0 × 10^4^ to 1.0 × 10^0^ copies/μL was used as templates.

### 2.8 Assessment of specificity

The total nucleic acids of the following viruses were used as templates to assess the specificity: genotypes I and II ASFV, PCV2, PRV, PRRSV, PEDV, FMDV, CSFV, and SIV. The nuclease-free distilled water, and negative tissue samples were used as negative controls.

### 2.9 Assessment of sensitivity

The mixtures of three plasmid constructs were 10-fold serially diluted. The mixtures with final reaction concentrations ranging from 1.0 × 10^5^ to 1.0 × 10^−2^ copies/μL were used as templates, and the limits of detection (LODs) were determined by Poisson distribution analysis.

In addition, the mixtures with final reaction concentrations ranging from 250 to 0.25 copies/reaction were used as templates, and the LODs were analyzed using PROBIT regression in SPSS 26.0 software,^2^ and the related figures were generated using Statacorp stata 17 software.^3^

### 2.10 Assessment of repeatability

To assess the repeatability, the mixtures of three plasmid constructs at final concentrations of 1.0 × 10^4^, 1.0 × 10^3^, and 1.0 × 10^2^ copies/μL were used to perform the intra-assay and inter-assay tests, and the coefficients of variation (CVs) were calculated.

### 2.11 Evaluation of the clinical samples

The developed triplex cdPCR, and the triplex qPCR reported by Qian et al. (23) were used to test 1,275 clinical samples obtained in Guangxi province of China. The clinical sensitivity and specificity of the triplex cdPCR were evaluated, and the agreement rates of the detection results between both methods were determined using SPSS 26.0 software (see footnote 2).

## 3 Results

### 3.1 Generation of the standard plasmids

The targeted fragments of the ASFV B646L, F1055L, and E183L genes were obtained by PCR amplification using the primers in Table 1, followed by purification and ligation into the pMD18-T vector, then transformed into *E. coli* DH5α competent cells. The positive clones were cultured, and the plasmid constructs were extracted. Finally, the concentrations of three standard plasmid constructs named p-dASFV-B646L, p-dASFV-F1055L, and p-dASFV-E183L were determined to be 3.69 × 10^10^, 1.81 × 10^10^, and 1.0 × 10^10^ copies/μL, respectively. All plasmid constructs were diluted to 1.0 × 10^10^ copies/μL, and stored at −80°C until used.

### 3.2 Determination of the reaction conditions

**Figure 1:**
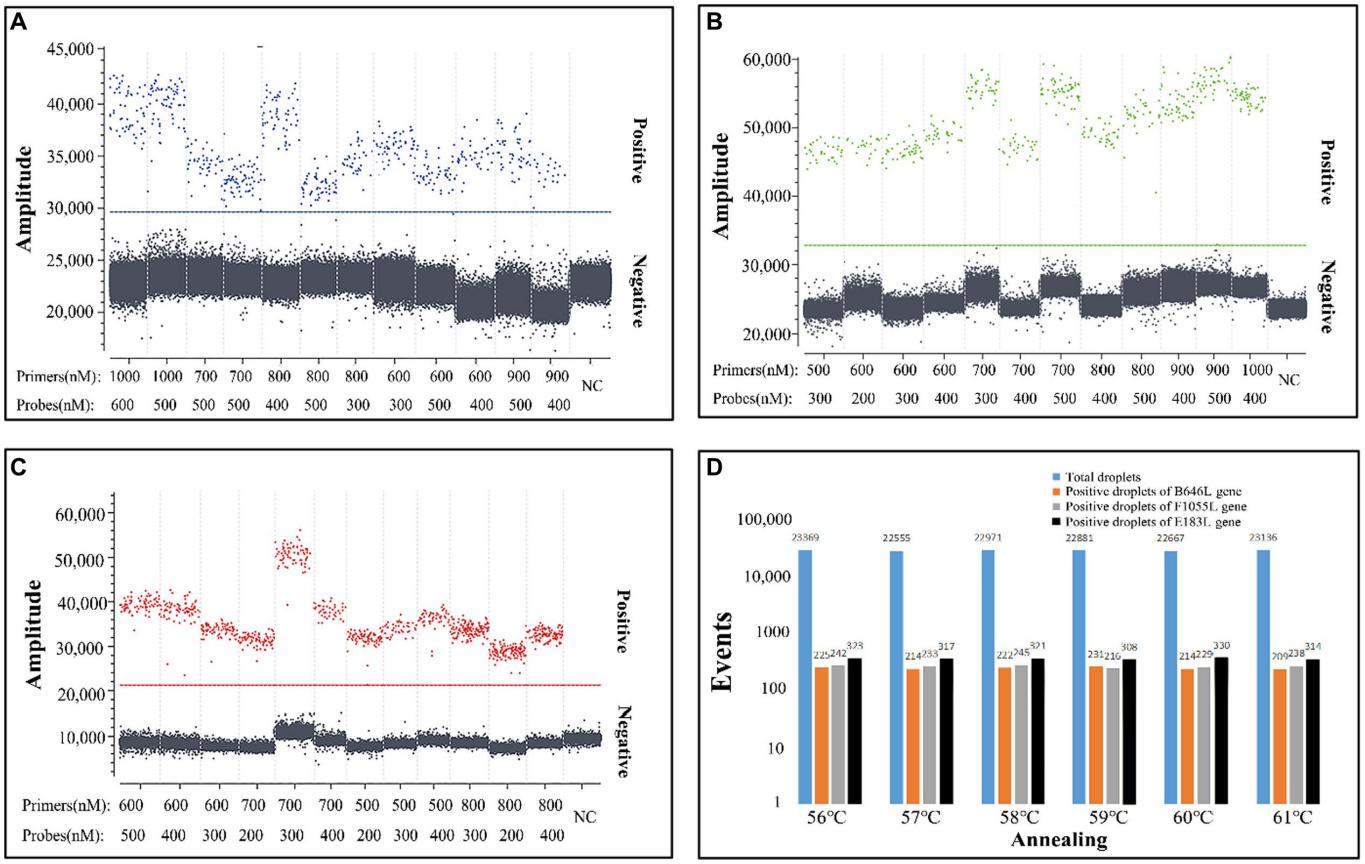
Optimization of the concentrations of primers and probes **(A–C)** and the annealing temperature **(D)**. The three plasmid constructs p-dASFV-B646L, p-dASFV-F1055L, and p-dASFV-E183L were mixed at the final concentrations of 1.0 × 10^2^ copies/μL. The amplification results of 12 different combinations of probe and primer concentrations are shown in panel **(A–C)**, and the amplification results at different annealing temperatures are shown in panel **(D)**. NC, Negative control.

The triplex cdPCR was developed using the Naica™ Sapphire Crystal System (Stilla Technologies™, Villejuif, France). After experiments on optimizing combinations of primers and probes at different concentrations, annealing temperatures, and reaction cycles, the optimal reaction conditions were obtained, and a triplex cdPCR was established (Figure 1). The reaction system contained PerfeCTa Multiplex qPCR ToughMix (Quanta Biosciences, Gaithersburg, MD, USA), Fluorescein Sodium Salt (1 μM) (Apexbio Biotechnology, Beijing, China), three primers and probes, the mixtures of three plasmid constructs, and nuclease-free water (Table 2). The amplification procedure: 95°C for 30 s, 45 cycles of 95°C for 5 s, and 56°C for 30 s. After amplification, the Sapphire chips (Stilla Technologies, France) were shifted into the Naica™ Prism3 (Stilla Technologies, France), and each sample’s absolute concentration were automatically reported with 3 high-resolution images.

**Table 2:** Reaction system of the triplex cdPCR.

Reagent	Volume (μL)	Final concentration (nM)
PerfeCTa Multiplex qPCR ToughMix (2×)	12.5	1×
Fluorescein Sodium Salt (1 μM)	2.5	100
B646L -F (25 μM)	0.8	800
B646L -R (25 μM)	0.8	800
B646L -P (25 μM)	0.4	400
F1055L-F (25 μM)	0.7	700
F1055L-R (25 μM)	0.7	700
F1055L-P (25 μM)	0.3	300
E183L-F (25 μM)	0.7	700
E183L-R (25 μM)	0.7	700
E183L-P (25 μM)	0.3	300
Total nucleic acids	2.5	/
Nuclease-free distilled water	Up to 25	/

### 3.3 Generation of the standard curves

**Figure 2:**
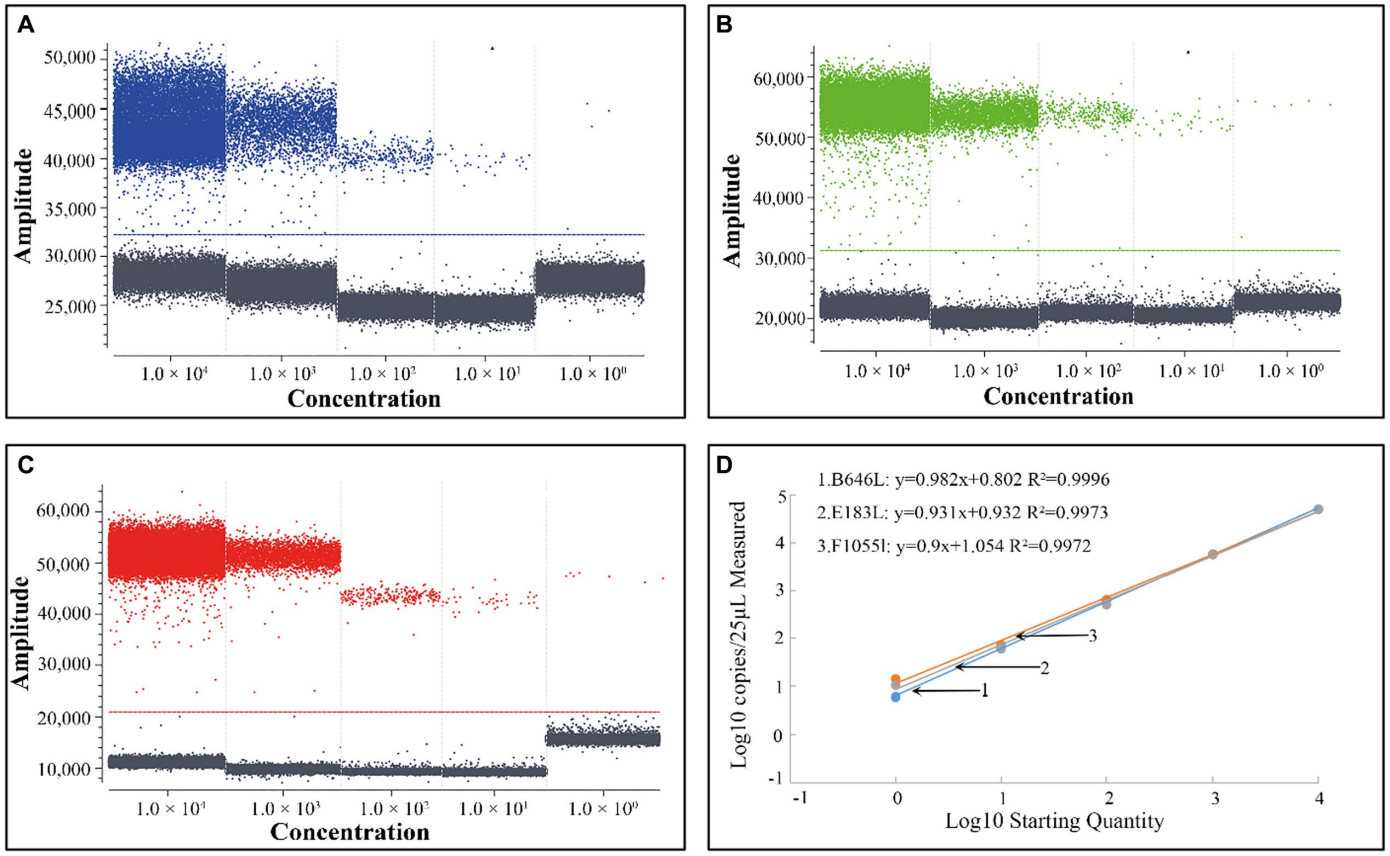
The positive droplets of the plasmid constructs p-dASFV-B646L **(A)**, p-dASFV- F1055L **(B)**, and p-dASFV- E183L **(C)** at different final concentrations from 1.0 × 10^4^ to 1.0 × 10^0^ copies/μL and the standard curves **(D)**.

To obtain the standard curves of the triplex cdPCR, the standard plasmid constructs p-dASFV-B646L, p-dASFV-F1055L, and p-dASFV-E183L at final concentrations from 1.0 × 10^4^ to 1.0 × 10^0^ copies/μL were used as templates. The results showed that the slopes and R^2^ were 0.982 and 0.9996, 0.9 and 0.9972, and 0.931 and 0.9973, for the B646L, F1055L, and E183L genes, respectively (Figure 2).

### 3.4 Specificity analysis

**Figure 3:**
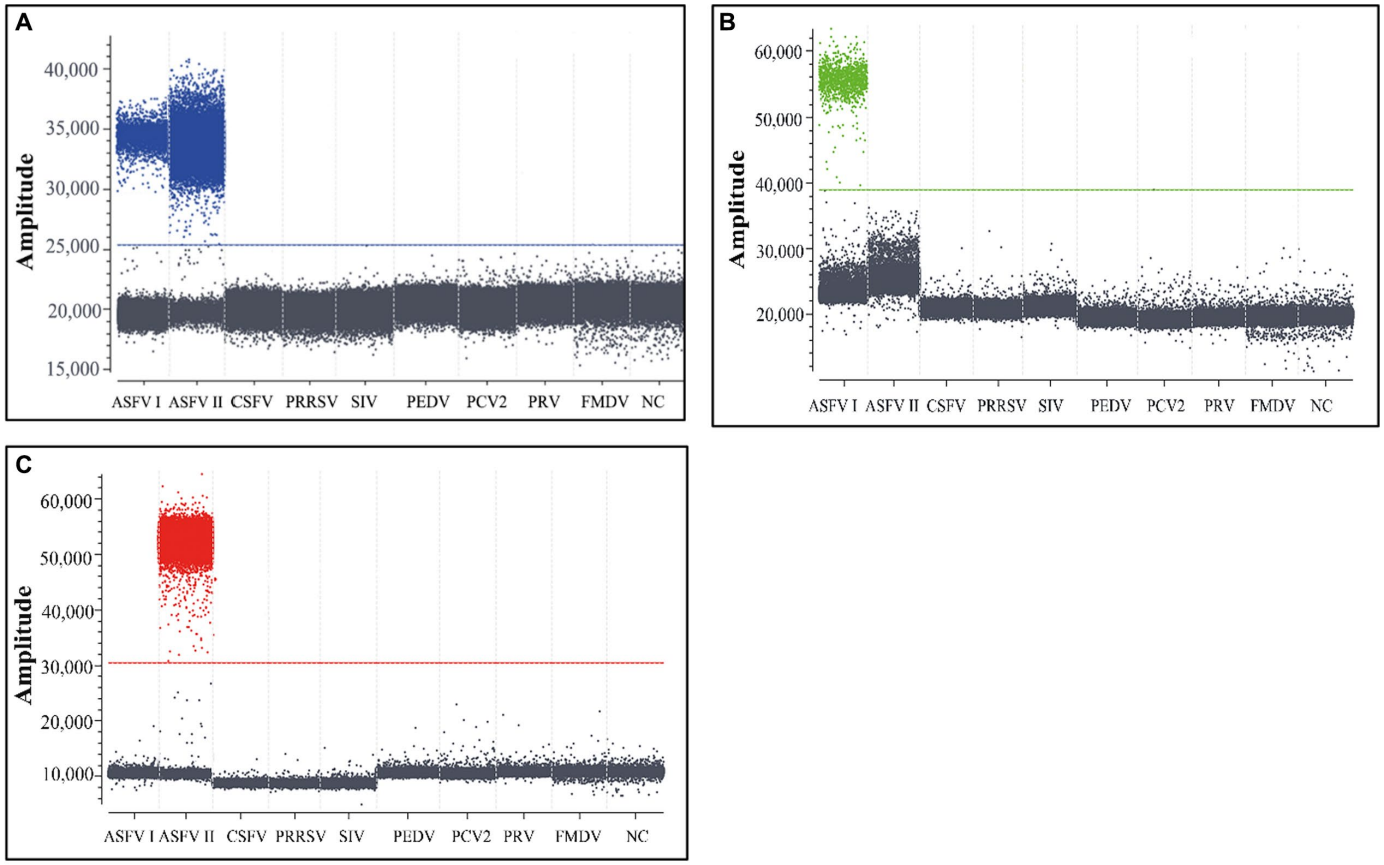
The specificity analysis of the triplex cdPCR **(A–C)**. The results for B646L gene **(A)**, F1055L gene **(B)**, and E183L gene **(C)** are showed, respectively. Genotype I ASFV, genotype II ASFV, PCV2, PRV, PRRSV, PEDV, FMDV, CSFV, and SIV were used to analyze the triplex cdPCR’s specificity. NC, Negative control.

The total nucleic acids of genotype I ASFV, genotype II ASFV, PCV2, PRV, PRRSV, PEDV, FMDV, CSFV, and SIV were used to analyze the triplex cdPCR’s specificity. The results showed that the positive droplets could only obtained from genotypes I and II ASFV, but not from the other porcine viruses (Figure 3).

### 3.5 Sensitivity analysis

**Figure 4:**
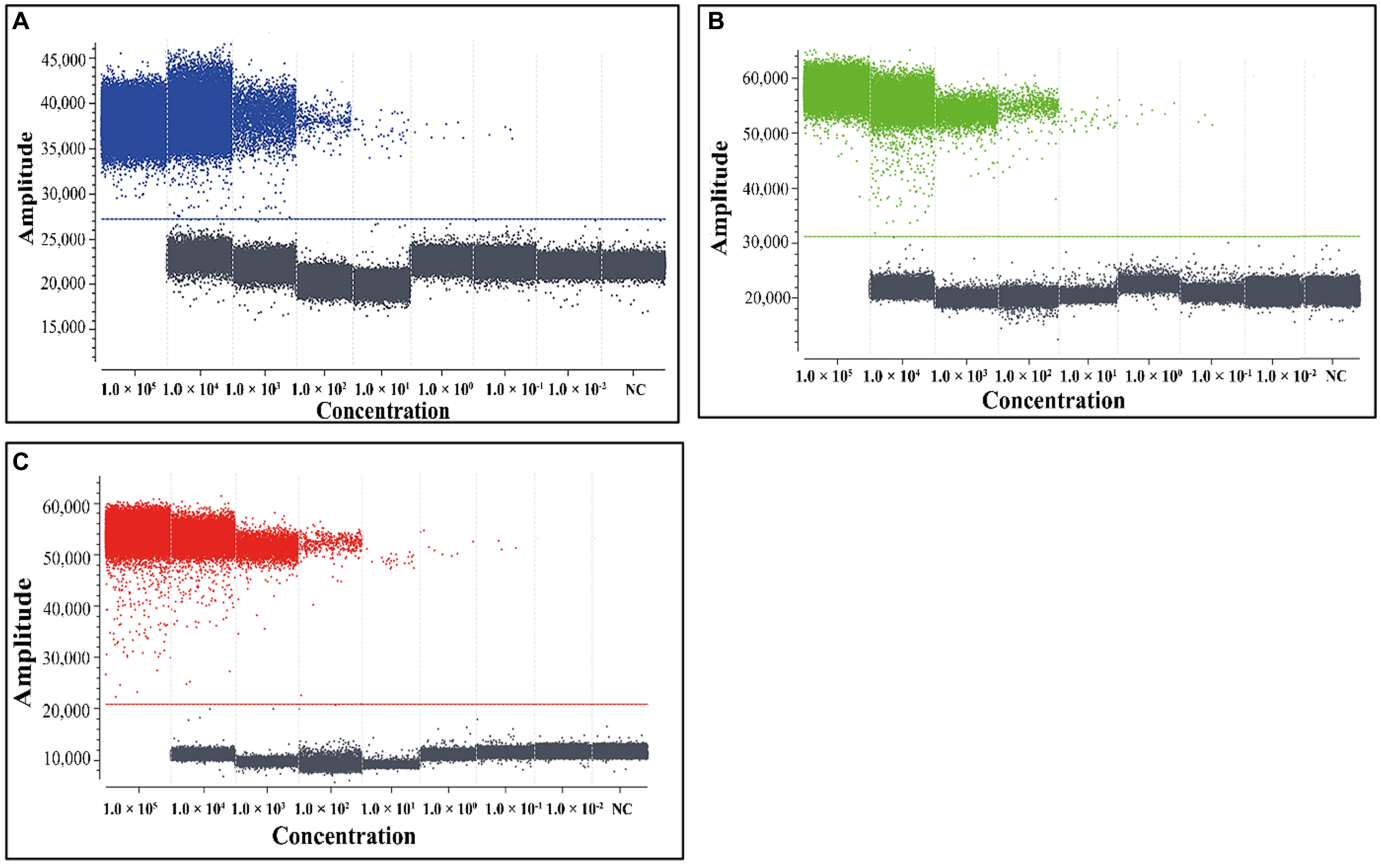
The sensitivity analysis of the triplex cdPCR **(A–C)**. The plasmid constructs p-dASFV-B646L **(A)**, p-dASFV-F1055L **(B)**, and p-dASFV-E183L **(C)** are used for sensitivity analysis, respectively. The mixtures of three plasmid constructs with final concentrations from 1.0 × 10^5^ to 1.0 × 10^−2^ copies/μL were used as templates. NC, Negative control.

In order to assess the LOD of the triplex cdPCR, the mixtures of three plasmid constructs p-dASFV-B646L, p-dASFV-F1055L, and p-dASFV-E183L from 1.0 × 10^5^ to 1.0 × 10^−2^ copies/μL (final concentration) were used as templates. The results showed that the number of positive droplets decreased in a gradient as the concentrations of the mixtures decreased. According to the Poisson distribution, the LODs of p-dASFV-B646L, p-dASFV-F1055L, and p-dASFV-E183L were 6.5, 4.5, and 5.75 copies/reaction, respectively (Figure 4).

**Figure 5:**
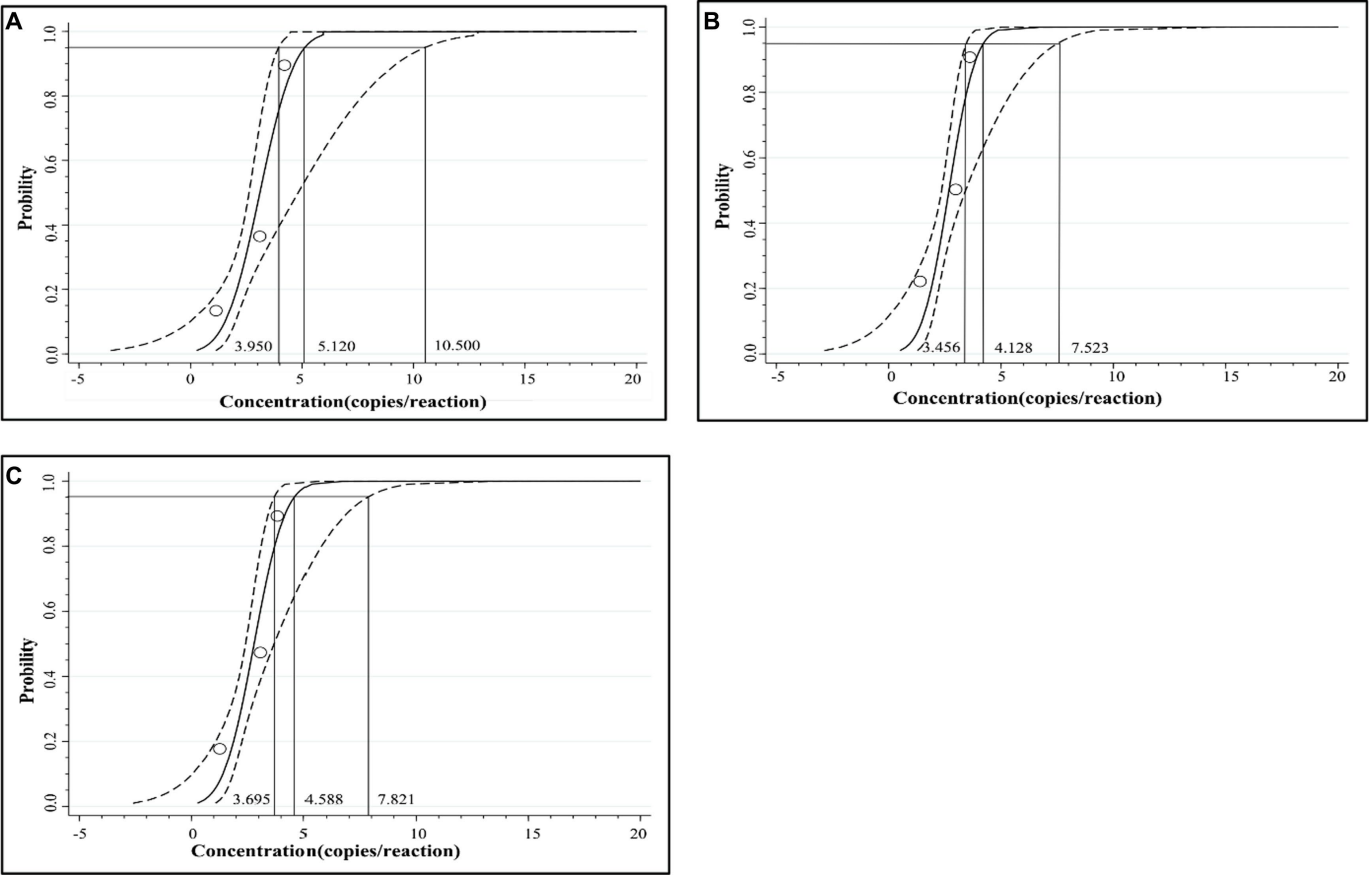
The results of PROBIT regression analysis for the triplex cdPCR. The LODs of p-dASFV-B646L **(A)**, p-dASFV-F1055L **(B)**, and p-dASFV-E183L **(C)** were determined to be 5.120 (3.950–10.500 at 95% CI), 4.218 (3.456–7.523 at 95% CI), and 4.588 (3.695–7.821 at 95% CI) copies/reaction, respectively.

The mixed three plasmid constructs, p-dASFV-B646L, p-dASFV-F1055L, and p-dASFV-E183L ranging from 250 to 0.25 copies/reaction (final concentration) were used to determine the number of positive droplets and the hit rates. The results are shown in Table 3. The LODs of the three plasmid constructs analyzing by PROBIT regression analysis showed that the LODs of p-dASFV-B646L, p-dASFV-F1055L, and p-dASFV-E183L were 5.120 (3.950–10.500 at 95% confidence interval (CI)), 4.218 (3.456–7.523 at 95% CI), and 4.588 (3.695–7.821 at 95% CI) copies/reaction, respectively (Figure 5).

**Table 3:** Number of positive samples and hit rates for serial dilution of plasmid constructs.

Plasmid construct	Copies/Reaction	Number of sample	Positive sample	Hit rate (%)
dASFV-B646L	250	48	48	100
	25	48	48	100
	2.5	48	14	29.2
	0.25	48	0	0
dASFV-F1055L	250	48	48	100
	25	48	48	100
	2.5	48	20	41.7
	0.25	48	0	0
dASFV-E183L	250	48	48	100
	25	48	48	100
	2.5	48	18	37.5
	0.25	48	0	0

### 3.6 Repeatability analysis

The mixtures of three plasmid constructs with the final concentration of 10^4^, 10^3^, and 10^2^ copies/μL were used as templates. The results showed that the intra-assay CVs were 1.24–2.01%, and the inter-assay CVs were 1.30–2.53% (Table 4).

**Table 4:** Repeatability assessment of the triplex cdPCR.

Plasmid	Concentration (Copies/μL)	Ct value of intra-assay			Ct value of inter-assay		
		^−^x (Copies/Reaction)	SD	CV (%)	^−^x (Copies/Reaction)	SD	CV (%)
p-dASFV-B646L	1.0 × 10^4^	34816.67	464.58	1.33	34841.67	488.83	1.40
	1.0 × 10^3^	3256.67	40.41	1.24	3283.33	62.92	1.91
	1.0 × 10^2^	320	5	1.56	335.67	7.51	2.36
p-dASFV-F1055L	1.0 × 10^4^	39,000	595.29	1.53	39,275	513	1.30
	1.0 × 10^3^	3250.83	48.76	1.50	3239.17	42.90	1.32
	1.0 × 10^2^	304.17	3.82	1.26	315.83	6.30	2.02
p-dASFV-E183L	1.0 × 10^4^	35,850	537.94	1.50	36391.67	619.64	1.70
	1.0 × 10^3^	3408.33	52.70	1.55	3462.33	52.79	1.52
	1.0 × 10^2^	327.50	6.61	2.01	337	8.54	2.53

### 3.7 Testing of the clinical samples

**Figure 6:**
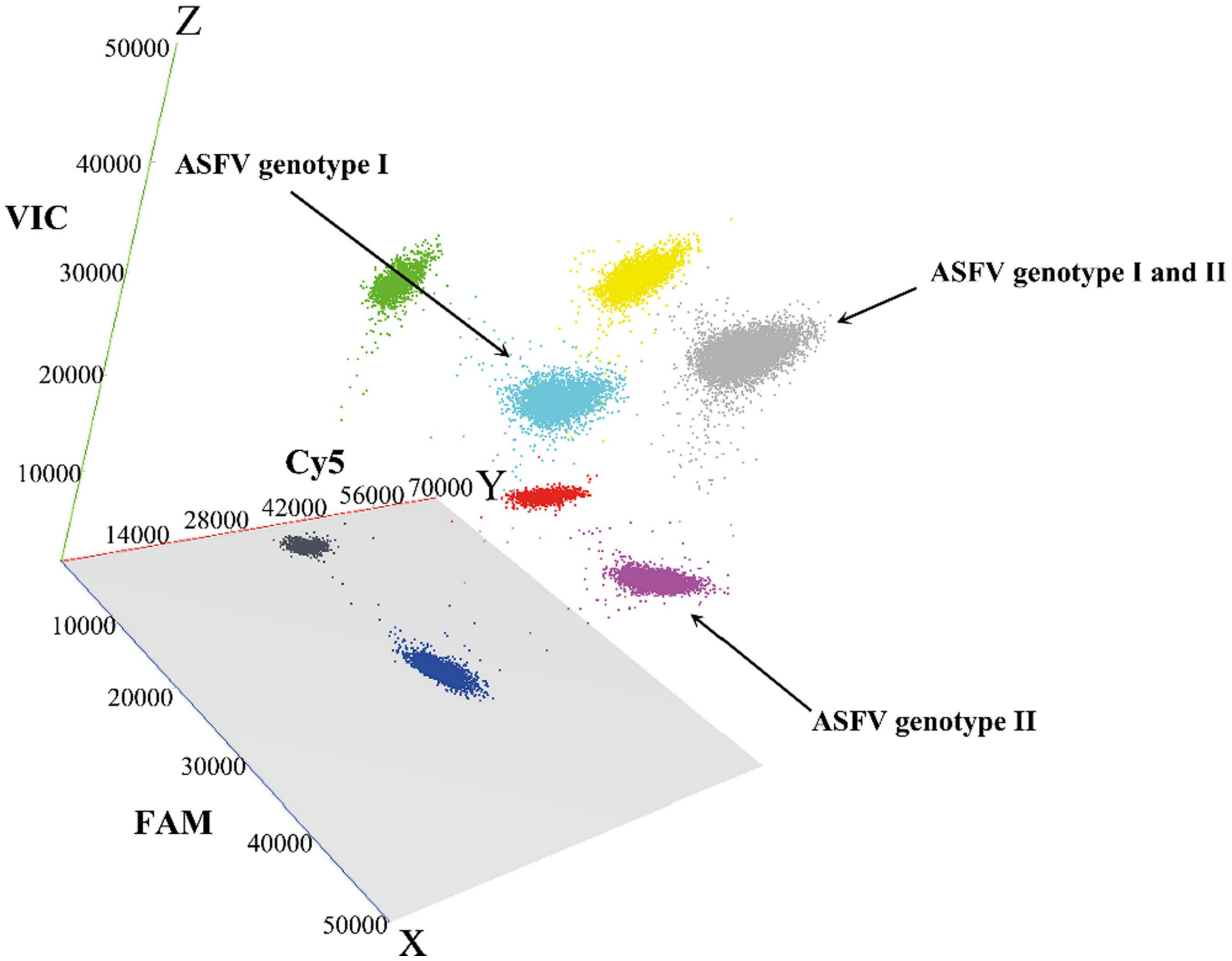
The 3D scatterplots of fluorescence intensity for the clinical samples. The data were obtained in the FAM (blue), Cy5 (red), and VIC (green) acquisition channels.

The established triplex cdPCR were used to evaluate the 1,275 clinical samples from Guangxi province. The results showed that a total of 118 (9.25%, 118/1,275) ASFV-positive samples were detected, including 64 (5.02%, 64/1,275) positive samples of genotype I, 41 (3.22%, 41/1,275) positive samples of genotype II, and 13 (1.02%, 13/1,275) positive samples of co-infection with genotypes I and II (Table 5). The 3D dot plots are shown to display the data of co-infections in clinical samples through using three-dimensional scatterplots to allow immediate visualization (Figure 6).

**Table 5:** The results of clinical samples using the developed triplex cdPCR.

Source	Total	Positive	Genotype I	Genotype II	Genotype I and II	Positive rate
Pig farm	98	2	0	0	2	2.04% (2/98)
Harmless disposal site	632	96	61	26	9	15.19% (96/632)
Slaughterhouse	545	20	3	15	2	3.67% (20/545)
Total	1,275	118 (9.25%)	64 (5.02%)	41 (3.22%)	13 (1.02%)	9.25% (118/1,275)

The 1,275 clinical samples were also tested using the triplex qPCR reported by Qian et al. (23). The results showed that a total of 105 (8.24%, 105/1,275) ASFV-positive samples were detected, including 58 (4.55%, 58/1,275) positive samples of genotype I, 36 (2.82%, 36/1,275) positive samples of genotype II, and 11 (0.86%, 11/1,275) positive samples of co-infection with genotypes I and II (Table 6). In addition, the clinical sensitivity and clinical specificity of the triplex cdPCR were 100, and 98.89%, respectively (Table 7). The agreements between the triplex cdPCR and the triplex qPCR were more than 98.98% (Table 6).

**Table 6:** The agreements of the triplex cdPCR and the reported reference triplex qPCR.

Pathogen	Number	Triplex qPCR		Triplex cdPCR		Agreement rate (%)	Kappa
		Positive	Positive rate (%)	Positive	Positive rate (%)		
Genotype I	1,275	58	4.55	64	5.02	99.53	0.948
Genotype II	1,275	36	2.82	41	3.22	99.61	0.933
Genotypes I and II	1,275	11	0.86	13	1.02	99.84	0.916
Total	1,275	105	8.24	118	9.25	98.98	0.936

Positive percent agreement (PPA): 100% (96.47–100% at 95% CI).

Negative percent agreement (NPA): 98.89% (98.11–99.35% at 95% CI).

Overall percent agreement (OPA): 98.98% (98.26–99.40% at 95% CI).

**Table 7:** The clinical sensitivity and specificity of the triplex cdPCR.

Triplex cdPCR	Triplex qPCR		Total	Clinical Sensitivity	Clinical Specificity
	Positive	Negative			
Positive	105	13	118	100%	98.89%
Negative	0	1,157	1,157		
Total	105	1,170	1,275		

Clinical sensitivity: 100% (96.47–100% at 95% CI).

Clinical specificity: 98.89% (98.11–99.35% at 95% CI).

## 4 Discussion

ASF has caused significant economic losses to domestic pigs and wild boars due to the disease’s acute course and high mortality rate (2). The World Organization for Animal Health (WOAH) identifies ASF as one of the listed diseases. ASF was endemic within the African continent prior to 1957, but spread outside this continent thereafter. The highly pathogenetic genotype II ASF was first outbroke in China in 2018 (7), and relatively lower virulent genotype I ASFV was first discovered in 2020 (21). Many Asian countries has been reported ASFV since 2018 (5, 10, 11). To date, ASF has been founded in a multitude of countries in Africa, Asia, Europe, and America (10–14). Even if ASF is epidemic worldwide, genotype I and II ASFV were the only two genotypes of the 24 genotypes of ASFV that have ever been identified outside of African continent until now (16–18). However, due to the even increasement of global trade and the volume of imported and exported animals and animal’s products, there is always a risk of ASFV spilling from the African continent to other continents (37–39). Development of a rapid, reliable, and accurate method for detection, surveillance, and diagnosis of ASFV is very urgent for the countries where ASF is circulating. The qPCR has been extensively used to detect viral nucleic acids in many laboratories, since this technique is known for its high sensitivity, excellent specificity, and reliability. It allows for the accurate and efficient identification of viral infections, enabling timely and targeted interventions for disease’s prevention and control (29, 30). Several reports have developed qPCR to detect ASFV and distinguish genotypes I and II ASFV (23, 40–43). However, the qPCR has the disadvantages of the fluctuation of Ct values depending on threshold setting, the high sensitivity to reaction inhibitors, and the complex procedure of generating calibration curves. Therefore, the dPCR is a new and better choice for detection of viral nucleic acids. The dPCR has the advantages of absolute quantification of template independent on the Ct values and standard curves, the excellent sensitivity and precision for low loads of templates, and low sensitivity to PCR inhibitors. Several reports have established the dPCR for detection of ASFV (32–36), but no dPCR to simultaneously detect genotypes I and II ASFV has been established. In this study, a triplex cdPCR was developed to detect and differentiate genotype I and genotypes II ASFV. Besides the abovementioned advantages of the cdPCR, the multiplex cdPCR can make full use of the apparatus to detect several viruses in one reaction at the same time, which decrease the fee of detection dramatically. According to our previous calculation, it costs about US $17.67/sample by the singleplex dPCR, US $7.86/sample by the multiplex dPCR, and US $3.83/sample by the multiplex qRT-PCR [33]. This help the triplex cdPCR established in this study to apply for high-throughput detection of clinical samples, especially for the low viral-load clinical samples.

In this study, three pairs of specific primers and corresponding probes were designed basing on the B646L gene, F1055L gene, and E183L gene, respectively. The primers and probe targeting the B646L gene was used as universal primers and probe to detect 24 genotypes of ASFV. The synthesized plasmid constructs of 24 genotypes ASFV have been used to validate and confirm the viability of the primers and probe (23). The primers and probe targeting the F1055L gene was used to specifically amplify genotype I ASFV, and the primers and probe targeting the E183L gene was used to specifically amplify genotype II ASFV. After optimizing the reaction parameters, such as primer and probe concentrations, annealing temperatures, and reaction cycles, a triplex cdPCR was successfully developed. The assay achieved remarkable specificity, ensuring that only the targeted viral nucleic acids of ASFV were amplified and detected. The sensitivity of the assay has been greatly enhanced, obtaining the LODs of 5.120, 4.218, and 4.588 copies/reaction for the B646L, F1055L, and E183L genes, respectively, while the multiplex qPCR using the same primers and probe had the LODs of 399.647, 374.409, 355.083 copies/reaction for the B646L, F1055L, and E183L genes, respectively (23), indicating that the triplex cdPCR had 78.06, 88.76, 77.39 times higher than those of the triplex qPCR, respectively. The excellent sensitivity of the assay enables it to detect very low viral loads, which is crucial for the early stage of infection. Repeatability analysis of the assay was excellent, with the intra-assay and inter-assay CVs between 1.24 and 2.53%. The R^2^ values of the standard curves were ≥ 0.997, indicating a good linear relationship between the initial templates and the positive droplet values. The cdPCR method offers a notably lower LODs in comparison to the qPCR (23), making it more suitable for evaluating the clinical samples with low viral loads. The triplex cdPCR had clinical sensitivity and specificity of 100, and 98.89%, respectively, and had agreements of more than 98.98% with the reference triplex qPCR when they were used to evaluate the 1,275 clinical samples. These advancements have significantly enhanced the accuracy and reliability of the developed triplex cdPCR for detection of viral nucleic acids.

The developed triplex cdPCR was used to evaluate 1,275 clinical samples collected between March 2023 and August 2023 in Guangxi province. The positivity rates of genotype I, genotype II, and co-infection of genotypes I + II were 5.02, 3.22, and 1.02%, respectively, with a total positivity rate of 9.25% in clinical samples, indicating that ASFV is still epidemic in Guangxi province. However, compared to the previous data reported in Guangxi province (19, 22, 23, 33, 36, 44–46), the positivity rate of ASFV in Guangxi province in this study was significant decreased. In Guangxi province of China, the reported positivity rates of ASFV in clinical samples collected during different periods were as follows: 57.14% (192/336) from January 2019 to December 2020 (19), 45.58% (232/509) from October 2018 to December 2020 (22), 43.75% (168/384) from October 2018 to December 2019 (44), 30.10% (87/289) from January 2018 to March 2021 (33), 25.63% (293/1,143) from February 2018 to March 2021 (45), 14.17% (214/1,510) from January 2022 to December 2022 (36), 12.60% (534/4,239) from January 2021 to December 2021 (46), 8.16% (287/3,519) from March 2019 to February 2023 (23), 9.25% (118/1,275) from March 2023 to August 2023 (this study). Overall, the positivity rates of ASFV in Guangxi province have gradually decreasing since 2018, the year ASF first outbroke in China. In addition, it is noteworthy that the positivity rate of genotype I was higher than that of genotype II in this study, whereas genotype II was the predominant genotype in the previous reports (19, 22, 23, 33, 36, 44–46), indicating that genotype I might have become the main circulating genotype in Guangxi province in 2023, which needs to be further confirmed through larger and longer epidemiological investigations. Unfortunately, there are few reports on the monitoring results of ASFV in various provinces of China, so we cannot know the current epidemic situation in various regions. The decrease in the prevalence of ASFV in Guangxi province suggests that the prevention and control measures carried out in China were very effective. The main measures included strict biosecurity, accurate detection and rapid diagnosis, rule out the ASFV-positive pigs in the very early infected stage (47, 48). Therefore, a rapid, sensitive, and accurate method to detect ASFV is vital in order to accurately identify the early-stage infected pigs, and decisively clear them at designated points. This assay can be used to accurately and efficiently detect genotypes I and II strains of ASFV, allowing for rapid and targeted interventions to prevent further spread and mitigate the impact on pig populations.

## 5 Conclusion

A rapid, sensitive, and accurate triplex cdPCR was developed to detect and differentiate genotype I, and genotype II ASFV. The highly sensitive, specific, and reproducible assay is suitable for detection and investigation of ASFV in clinical samples. In addition, the genotype I strains of ASFV is the important circulating strains besides the genotype II strains in Guangxi province in China at present.

## Data availability statement

The raw data supporting the conclusions of this article will be made available by the authors, without undue reservation.

## Ethics statement

This study was approved by Guangxi Center for Animal Disease Control and Prevention (CADC) (No. 2020-A-01). Guangxi CADC was approved by the Ministry of Agriculture and Rural Affairs, China for collection and detection of ASFV in clinical samples (Approval Number: 2018-154-25). The study was conducted in accordance with the local legislation and institutional requirements.

## Author contributions

KS: Writing – review & editing, Funding acquisition. XQ: Writing – original draft, Methodology. YS: Writing – original draft, Methodology. HW: Writing – original draft, Validation, Investigation. YP: Writing – original draft, Software, Data curation. FL: Writing – original draft, Project administration. QZ: Writing – original draft, Visualization. SM: Writing – review & editing, Project administration. LH: Writing – original draft, Validation, Investigation. ZL: Writing – review & editing, Funding acquisition.

## Footnotes

^1^ https://blast.ncbi.nlm.nih.gov/Blast.cgi

^2^ https://www.ibm.com/cn-zh/spss

^3^ https://www.stata.com/products/windows/

## References

[1] Wang G Xie M Wu W Chen Z. Structures and functional diversities of ASFV proteins. Viruses. (2021) 13:2124. doi: 10.3390/v13112124, PMID: 34834930 PMC8619059

[2] Wang F Zhang H Hou L Yang C Wen Y. Advance of African swine fever virus in recent years. Res Vet Sci. (2021) 136:535–9. doi: 10.1016/j.rvsc.2021.04.004, PMID: 33882382

[3] Galindo I Alonso C. African swine fever virus: a review. Viruses. (2017) 9:103. doi: 10.3390/v9050103, PMID: 28489063 PMC5454416

[4] Gaudreault NN Madden DW Wilson WC Trujillo JD Richt JA. African swine fever virus: An emerging DNA arbovirus. Front Vet Sci. (2020) 7:215. doi: 10.3389/fvets.2020.00215, PMID: 32478103 PMC7237725

[5] Xin G Kuang Q Le S Wu W Gao Q Gao H. Origin, genomic diversity and evolution of African swine fever virus in East Asia. Virus Evol. (2023) 9:vead060. doi: 10.1093/ve/vead060, PMID: 37868933 PMC10590196

[6] Penrith ML Van Heerden J Heath L Abworo EO Bastos ADS. Review of the pig-adapted African swine fever viruses in and outside Africa. Pathogens. (2022) 11:1190. doi: 10.3390/pathogens11101190, PMID: 36297247 PMC9609104

[7] Zhou X Li N Luo Y Liu Y Miao F Chen T. Emergence of African swine fever in China, 2018. Transbound Emerg Dis. (2018) 65:1482–4. doi: 10.1111/tbed.12989, PMID: 30102848

[8] Tao D Sun D Liu Y Wei S Yang Z An T. One year of African swine fever outbreak in China. Acta Trop. (2020) 211:105602. doi: 10.1016/j.actatropica.2020.105602, PMID: 32598922

[9] Zhou L Yu EYW Wang S Sun C. African swine fever epidemic in China. Vet Rec. (2019) 184:713. doi: 10.1136/vr.l4026, PMID: 31175249

[10] Mighell E Ward MP. African swine fever spread across Asia, 2018–2019. Transbound Emerg Dis. (2021) 68:2722–32. doi: 10.1111/tbed.14039, PMID: 33599077

[11] Dixon LK Stahl K Jori F Vial L Pfeiffer DU. African swine fever epidemiology and control. Annu Rev Anim Biosci. (2020) 8:221–46. doi: 10.1146/annurev-animal-021419-083741 31743062

[12] Gonzales W Moreno C Duran U Henao N Bencosme M Lora P. African swine fever in the Dominican Republic. Transbound Emerg Dis. (2021) 68:3018–9. doi: 10.1111/tbed.14341, PMID: 34609795

[13] Sánchez-Cordón PJ Montoya M Reis AL Dixon LK. African swine fever: a re-emerging viral disease threatening the global pig industry. Vet J. (2018) 233:41–8. doi: 10.1016/j.tvjl.2017.12.025, PMID: 29486878 PMC5844645

[14] Ata EB Li ZJ Shi CW Yang GL Yang WT Wang CF. African swine fever virus: a raised global upsurge and a continuous threaten to pig husbandry. Microb Pathog. (2022) 167:105561. doi: 10.1016/j.micpath.2022.105561, PMID: 35526679

[15] Danzetta ML Marenzoni ML Iannetti S Tizzani P Calistri P Feliziani F. African swine fever: lessons to learn from past eradication experiences. A systematic review. Front Vet Sci. (2020) 7:296. doi: 10.3389/fvets.2020.00296, PMID: 32582778 PMC7296109

[16] Achenbach JE Gallardo C Nieto-Pelegrín E Rivera-Arroyo B Degefa-Negi T Arias M. Identification of a new genotype of African swine fever virus in domestic pigs from Ethiopia. Transbound Emerg Dis. (2017) 64:1393–404. doi: 10.1111/tbed.12511, PMID: 27211823

[17] Qu H Ge S Zhang Y Wu X Wang Z. A systematic review of genotypes and serogroups of African swine fever virus. Virus Genes. (2022) 58:77–87. doi: 10.1007/s11262-021-01879-0, PMID: 35061204 PMC8778497

[18] Gallardo C Casado N Soler A Djadjovski I Krivko L Madueño E. A multi gene-approach genotyping method identifies 24 genetic clusters within the genotype II-European African swine fever viruses circulating from 2007 to 2022. Front Vet Sci. (2023) 10:1112850. doi: 10.3389/fvets.2023.1112850, PMID: 36761884 PMC9905734

[19] Shi K Liu H Yin Y Si H Long F Feng S. Molecular characterization of African swine fever virus from 2019-2020 outbreaks in Guangxi province, southern China. Front Vet Sci. (2022) 9:912224. doi: 10.3389/fvets.2022.912224, PMID: 35782548 PMC9240437

[20] Ito S Bosch J Martínez-Avilés M Sánchez-Vizcaíno JM. The evolution of African swine fever in China: a global threat? Front Vet Sci. (2022) 9:828498. doi: 10.3389/fvets.2022.828498, PMID: 35425825 PMC9001964

[21] Sun E Huang L Zhang X Zhang J Shen D Zhang Z. Genotype I African swine fever viruses emerged in domestic pigs in China and caused chronic infection. Emerg Microbes Infect. (2021) 10:2183–93. doi: 10.1080/22221751.2021.1999779, PMID: 34709128 PMC8635679

[22] Liu H Shi K Zhao J Yin Y Chen Y Si H. Development of a one-step multiplex qRT–PCR assay for the detection of African swine fever virus, classical swine fever virus and atypical porcine pestivirus. BMC Vet Res. (2022) 18:43. doi: 10.1186/s12917-022-03144-4, PMID: 35042532 PMC8764768

[23] Qian X Hu L Shi K Wei H Shi Y Hu X. Development of a triplex real-time quantitative PCR for detection and differentiation of genotypes I and II African swine fever virus. Front Vet Sci. (2023) 10:1278714. doi: 10.3389/fvets.2023.1278714, PMID: 37929278 PMC10620837

[24] Zhao D Sun E Huang L Ding L Zhu Y Zhang J. Highly lethal genotype I and II recombinant African swine fever viruses detected in pigs. Nat Commun. (2023) 14:3096. doi: 10.1038/s41467-023-38868-w, PMID: 37248233 PMC10226439

[25] Gao X Liu T Liu Y Xiao J Wang H. Transmission of African swine fever in China through legal trade of live pigs. Transbound Emerg Dis. (2021) 68:355–60. doi: 10.1111/tbed.13681 32530109

[26] Bustin SA Mueller R. Real-time reverse transcription PCR (qRT-PCR) and its potential use in clinical diagnosis. Clin Sci (Lond). (2005) 109:365–79. doi: 10.1042/CS20050086 16171460

[27] Kralik P Ricchi M. A basic guide to real time PCR in microbial diagnostics: definitions, parameters, and everything. Front Microbiol. (2017) 8:108. doi: 10.3389/fmicb.2017.00108, PMID: 28210243 PMC5288344

[28] Kuypers J Jerome KR Kraft CS. Applications of digital PCR for clinical microbiology. J Clin Microbiol. (2017) 55:1621–8. doi: 10.1128/JCM.00211-17, PMID: 28298452 PMC5442518

[29] Kojabad AA Farzanehpour M Galeh HEG Dorostkar R Jafarpour A Bolandian M. Droplet digital PCR of viral DNA/RNA, current progress, challenges, and future perspectives. J Med Virol. (2021) 93:4182–97. doi: 10.1002/jmv.26846, PMID: 33538349 PMC8013307

[30] Tan LL Loganathan N Agarwalla S Yang C Yuan W Zeng J. Current commercial dPCR platforms: technology and market review. Crit Rev Biotechnol. (2023) 43:433–64. doi: 10.1080/07388551.2022.2037503, PMID: 35291902

[31] Madic J Zocevic A Senlis V Fradet E Andre B Muller S. Three-color crystal digital PCR. Biomol Detect Quantif. (2016) 10:34–46. doi: 10.1016/j.bdq.2016.10.002, PMID: 27990348 PMC5154636

[32] Jia R Zhang G Liu H Chen Y Zhou J Liu Y. Novel application of nanofluidic chip digital PCR for detection of African swine fever virus. Front Vet Sci. (2021) 7:621840. doi: 10.3389/fvets.2020.621840, PMID: 33614757 PMC7894257

[33] Shi K Chen Y Yin Y Long F Feng S Liu H. A multiplex crystal digital PCR for detection of African swine fever virus, classical swine fever virus, and porcine reproductive and respiratory syndrome virus. Front Vet Sci. (2022) 9:926881. doi: 10.3389/fvets.2022.926881, PMID: 35812859 PMC9270018

[34] Zhu J Jian W Huang Y Gao Q Gao F Chen H. Development and application of a duplex droplet digital polymerase chain reaction assay for detection and differentiation of EP402R-deleted and wild-type African swine fever virus. Front Vet Sci. (2022) 9:905706. doi: 10.3389/fvets.2022.905706, PMID: 35733636 PMC9207387

[35] Wu X Xiao L Lin H Chen S Yang M An W. Development and application of a droplet digital polymerase chain reaction (ddPCR) for detection and investigation of African swine fever virus. Can J Vet Res. (2018) 82:70–4. 29382972 PMC5764039

[36] Shi K Zhao K Wei H Zhou Q Shi Y Mo S. Triplex crystal digital PCR for the detection and differentiation of the wild-type strain and the MGF505-2R and I177L gene-deleted strain of African swine fever virus. Pathogens. (2023) 12:1092. doi: 10.3390/pathogens12091092, PMID: 37764900 PMC10534775

[37] Cheng J Ward MP. Risk factors for the spread of African swine fever in China: a systematic review of Chinese-language literature. Transbound Emerg Dis. (2022) 69:e1289–98. doi: 10.1111/tbed.14573, PMID: 35490407 PMC9790558

[38] Kedkovid R Sirisereewan C Thanawongnuwech R. Major swine viral diseases: An Asian perspective after the African swine fever introduction. Porcine Health Manag. (2020) 6:20. doi: 10.1186/s40813-020-00159-x, PMID: 32637149 PMC7336096

[39] Taylor RA Condoleo R Simons RRL Gale P Kelly LA Snary EL. The risk of infection by African swine fever virus in European swine through boar movement and legal trade of pigs and pig meat. Front Vet Sci. (2020) 6:486. doi: 10.3389/fvets.2019.00486, PMID: 31998765 PMC6962172

[40] Cao S Lu H Wu Z Zhu S. A duplex fluorescent quantitative PCR assay to distinguish the genotype I and II strains of African swine fever virus in Chinese epidemic strains. Front Vet Sci. (2022) 9:998874. doi: 10.3389/fvets.2022.998874, PMID: 36213412 PMC9539676

[41] Li X Hu Y Liu P Zhu Z Liu P Chen C. Development and application of a duplex real-time PCR assay for differentiation of genotypes I and II African swine fever viruses. Transbound Emerg Dis. (2022) 69:2971–9. doi: 10.1111/tbed.14459, PMID: 35061937

[42] Ilya T Monoldorova S Kang SS Yun S Byeon HS Mariia N. Development of a real-time recombinase polymerase amplification assay for the rapid detection of African swine fever virus genotype I and II. Pathogens. (2022) 11:439. doi: 10.3390/pathogens11040439, PMID: 35456114 PMC9026452

[43] Gao Q Feng Y Yang Y Luo Y Gong T Wang H. Establishment of a dual real-time PCR assay for the identification of African swine fever virus genotypes I and II in China. Front Vet Sci. (2022) 9:882824. doi: 10.3389/fvets.2022.882824, PMID: 35720851 PMC9198542

[44] Liu H Shi K Sun W Zhao J Yin Y Si H. Development a multiplex RT-PCR assay for simultaneous detection of African swine fever virus, classical swine fever virus and atypical porcine pestivirus. J Virol Methods. (2021) 287:114006. doi: 10.1016/j.jviromet.2020.114006, PMID: 33127443

[45] Chen Y Shi K Liu H Yin Y Zhao J Long F. Development of a multiplex qRT-PCR assay for detection of African swine fever virus, classical swine fever virus and porcine reproductive and respiratory syndrome virus. J Vet Sci. (2021) 22:e87. doi: 10.4142/jvs.2021.22.e87, PMID: 34854269 PMC8636662

[46] Zhao K Shi K Zhou Q Xiong C Mo S Zhou H. The development of a multiplex real-time quantitative PCR assay for the differential detection of the wild-type strain and the MGF505-2R, EP402R and I177L gene-deleted strain of the African swine fever virus. Animals. (2022) 12:1754. doi: 10.3390/ani12141754, PMID: 35883301 PMC9311895

[47] Gao L Sun X Yang H Xu Q Li J Kang J. Epidemic situation and control measures of African swine fever outbreaks in China 2018–2020. Transbound Emerg Dis. (2021) 68:2676–86. doi: 10.1111/tbed.13968, PMID: 33369865

[48] Liu Y Zhang X Qi W Yang Y Liu Z An T. Prevention and control strategies of African swine fever and progress on pig farm repopulation in China. Viruses. (2021) 13:2552. doi: 10.3390/v13122552, PMID: 34960821 PMC8704102

